# Treatment outcomes of *Mycobacterium avium* complex pulmonary disease according to disease severity

**DOI:** 10.1038/s41598-022-06022-z

**Published:** 2022-02-04

**Authors:** Bo-Guen Kim, Byung Woo Jhun, Hojoong Kim, O Jung Kwon

**Affiliations:** Division of Pulmonary and Critical Care Medicine, Department of Medicine, Samsung Medical Center, Sungkyunkwan University School of Medicine, 81 Irwon-ro, Gangnam-gu, Seoul, 06351 South Korea

**Keywords:** Respiratory tract diseases, Diseases

## Abstract

*Mycobacterium avium* complex pulmonary disease (MAC-PD) requires long-term treatment. We analyzed the outcomes of 992 MAC-PD patients according to disease severity and compared the outcomes of intermittent and daily therapy for mild disease. Patients were divided into groups according to severity using the body mass index, age, cavity, erythrocyte sedimentation rate, and sex (BACES) system, and culture conversion rates were evaluated. We also evaluated the effects of intermittent treatment on the culture conversion rates in mild disease group. Using the BACES, 992 patients were divided into mild (n = 331), moderate (n = 503), and severe (n = 158) disease groups, and culture conversion at the end of treatment was achieved in 85% (282/331), 80% (403/503), and 61% (97/158), respectively. Differences in culture conversion among the severity groups were significant (*p* < 0.001). In patients with mild disease, culture conversion rates were similar between intermittent (84%, 166/198) and daily (87%, 116/133) treatment (*p* = 0.396), and intermittent antibiotic therapy did not negatively impact culture conversion (adjusted hazard ratio 1.08; confidence interval 0.83–1.41; *p* = 0.578). MAC-PD patients with mild disease had higher culture conversion rates. Daily and intermittent therapy yielded similar culture conversion rates for mild disease. Treatment strategies with lower pill burden may be applicable in mild MAC-PD.

## Introduction

Nontuberculous mycobacteria (NTM) are ubiquitous organisms that cause chronic pulmonary disease (PD), and the burdens of this disease are increasing globally^1,2^. *Mycobacterium avium* complex (MAC), which is mainly composed of *M. avium* and *M. intracellulare*, is the most common pathogen^3,4^. Guidelines recommend treating MAC-PD patients with the combination of a macrolide, ethambutol, and rifamycin, with or without an injectable aminoglycoside, for at least 12 months after culture conversion^3,5^. However, a high pill burden and adverse drug effects frequently complicate long-term multidrug therapy^6^. Thus, it is not easy to maintain long-term antibiotics in real-world clinical practice, and yet, outcomes for MAC-PD are unsatisfactory^7–9^.

To reduce the pill burden without compromising the efficacy of the standard antibiotic treatment, studies have evaluated outcomes of a three-times-weekly intermittent therapy *versus* standard daily therapy^10–12^. A representative study found intermittent therapy to be effective for MAC-PD patients without cavitary lesions, high bacterial burden, or previous treatment^12^. Subsequent studies confirmed the effectiveness of intermittent therapy for nodular bronchiectatic (NB) PD without cavitary lesions^13,14^. Based on these data, recent guidelines recommend intermittent therapy for non-cavitary NB MAC-PD patients without advanced disease^3^. However, there is no consensus definition of “advanced” disease, and there is also a lack of tools for assessing the severity of NTM-PD.

Our research group recently evaluated the prognostic factors of NTM-PD by analyzing a study cohort with long-term follow-up data^15^. Subsequently, a disease severity scoring system, referred to as BACES [body mass index (BMI), age, cavity, erythrocyte sedimentation rate (ESR), and sex] was developed, which classified disease into three groups, i.e., mild, moderate, and severe^16^. In the present study, we compared the clinical characteristics and treatment outcomes among MAC-PD patients differing in disease severity. Subsequently, we compared the culture conversion rates between mild MAC-PD patients receiving intermittent or daily treatment.

## Results

### Baseline characteristics of study patients

**Figure 1:**
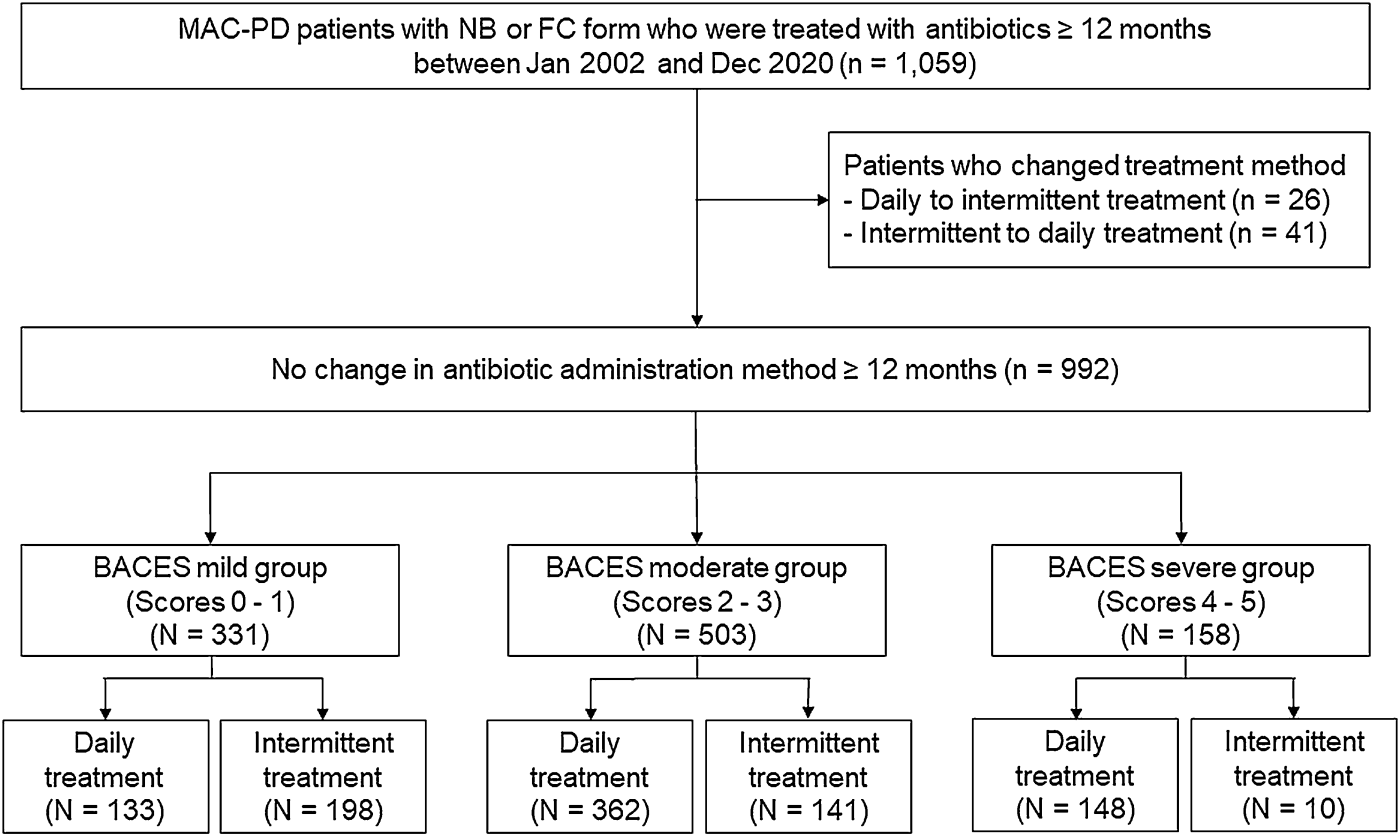
Study patients. Abbreviations: NB, nodular bronchiectatic; FC, fibrocavitary; BACES, BMI, age, cavity, ESR, and sex; BMI, body mass index.

Finally, 992 MAC-PD patients who were treated with antibiotics for ≥ 12 months without change in antibiotic administration method were included in this study (Fig. 1). Baseline characteristics of study patients are shown in Table 1. Overall, approximately a quarter (24%) of patients had a low BMI, and one-third (36%) were ≥ 65 years. Forty-five percent of patients had a cavity, and 38% were male. The most common underlying disease was previous pulmonary tuberculosis (42%), followed by obstructive pulmonary disease (12%), and chronic pulmonary aspergillosis (4%). More than half (52%) of patient had *M. intracellulare*-PD.

**Table 1:** Baseline characteristics of study patients according to BACES severity.

Variables	Total (n = 992)	Mild (n = 331)	Moderate (n = 503)	Severe (n = 158)	*p-*value
**BACES**					
BMI < 18.5 kg/m^2^	235 (24)	13 (4)	126 (25)	96 (41)	< 0.001^abc^
Age ≥ 65 years	356 (36)	17 (5)	204 (41)	135 (85)	< 0.001^abc^
Cavity	444 (45)	30 (9)	270 (54)	144 (91)	< 0.001^abc^
Elevated ESR*	719 (73)	135 (41)	431 (86)	153 (97)	< 0.001^abc^
ESR, mm/h	32 (18–56)	19 (11–31)	37 (23–64)	53 (35–80)	< 0.001^abc^
Sex, male	373 (38)	30 (9)	210 (42)	133 (84)	< 0.001^abc^
**Comorbidity**					
Previous pulmonary tuberculosis	413 (42)	99 (30)	217 (43)	97 (61)	< 0.001^abc^
Obstructive pulmonary disease	116 (12)	14 (4)	68 (14)	34 (22)	< 0.001^abc^
Chronic pulmonary aspergillosis	35 (4)	0 (0)	19 (4)	16 (10)	< 0.001^abc^
Lung cancer	22 (2)	4 (1)	8 (2)	10 (6)	0.001^bc^
Diabetes mellitus	73 (7)	14 (4)	42 (8)	17 (11)	0.017^c^
Chronic heart disease	58 (6)	10 (3)	35 (7)	13 (8)	0.023^ac^
Chronic kidney disease	12 (1)	5 (2)	5 (1)	2 (1)	0.730
Chronic liver disease	44 (4)	12 (4)	23 (5)	9 (6)	0.569
Cerebrovascular disease	29 (3)	5 (2)	17 (3)	7 (4)	0.120
Positive sputum AFB smear	482 (49)	101 (31)	264 (53)	117 (74)	< 0.001^abc^
Etiology					< 0.001^abc^
*Mycobacterium avium*	472 (48)	204 (62)	227 (45)	41 (26)	
*Mycobacterium intracellulare*	520 (52)	127 (38)	276 (55)	117 (74)	
Macrolide resistance^†^ (n = 891)	29/891 (3)	6/299 (2)	14/453 (3)	9/139 (7)	0.059
Radiological form					< 0.001^abc^
Nodular bronchiectatic form	747 (75)	323 (98)	376 (75)	48 (30)	
Non-cavitary	548 (55)	301 (91)	233 (46)	14 (9)	
Cavitary	199 (20)	22 (7)	143 (29)	34 (21)	
Fibrocavitary form	245 (25)	8 (2)	127 (25)	110 (70)	

Data are presented as n (%) or median (interquartile range).

*BACES* BMI, age, cavity, ESR, and sex, *BMI* body mass index, *ESR* erythrocyte sedimentation rate, *AFB* acid-fast bacilli.

*Elevated ESR: > 15 mm/h in men and > 20 mm/h in women.

^†^891 patients had information on macrolide susceptibility testing. ^a^*p* < 0.05 with Bonferroni correction between mild and moderate groups. ^b^*p* < 0.05 with Bonferroni correction between moderate and severe groups. ^c^*p* < 0.05 with Bonferroni correction between mild and severe groups.

Based on the BACES severity scores, 331 (33%) of study patients had mild disease, 503 (51%) had moderate, and 158 (16%) had severe disease (Table 1). The proportions of patients who had low BMI, old age, cavity, or elevated ESR, or who were males were significantly higher in the severe group compared to the mild or moderate severity groups (*p* < 0.001). Notably, most of mild group were not underweight (96%, ≥ 18.5 kg/m^2^) or not elderly (95%, < 65 year), and only 9% of the mild group had a cavitary lesion. The most common underlying disease was previous pulmonary tuberculosis in all severity groups. An increase in BACES severity was associated with a higher positive acid-fast bacilli (AFB) smear rate. Most (98%) of the mild group had the NB form on radiological findings, which was the highest proportion among the severity groups.

### Treatment outcome according to BACES severity

Treatment outcomes are shown in Table 2. The median treatment duration for all patients was 18.6 months [interquartile range (IQR), 15.3–24.1 months]. Seventy-five percent of all patients had culture conversion within 12 months of treatment, and 79% of all patients had achieved culture conversion by the end of treatment. Median time to culture conversion of the patients was 1.9 months (IQR 1.0–4.8) months. Patients with moderate or severe disease were more likely to receive adjunctive aminoglycoside injection or clofazimine in comparison with patients in the mild disease group.

**Table 2:** Treatment outcome according to BACES severity.

Variables	Total (n = 992)	Mild (n = 331)	Moderate (n = 503)	Severe (n = 158)	*p*-value
Total treatment duration, months	18.6 (15.3–24.1)	17.8 (15.1–23.7)	19.1 (15.2–24.1)	23.3 (17.9–26.5)	< 0.001^abc^
Culture conversion at 12 months	741 (75)	268 (81)	378 (75)	95 (60)	< 0.001^bc^
Culture conversion at the end of treatment	782 (79)	282 (85)	403 (80)	97 (61)	< 0.001^bc^
Time to culture conversion, months	1.9 (1.0–4.8)	1.2 (0.9–3.1)	2.3 (1.0–5.7)	3.1 (1.5–5.9)	< 0.001^ac^
Use of aminoglycoside injection	344 (35)	57 (17)	190 (38)	97 (61)	< 0.001^abc^
Use of clofazimine	49 (5)	7 (2)	29 (6)	13 (8)	0.007^abc^

Data are presented as n (%) or median (interquartile range).

*BACES* body mass index, age, cavity, erythrocyte sedimentation rate, and sex.

^a^*p* < 0.05 with Bonferroni correction between mild and moderate groups.

^b^*p* < 0.05 with Bonferroni correction between moderate and severe groups.

^c^*p* < 0.05 with Bonferroni correction between mild and severe groups.

**Figure 2:**
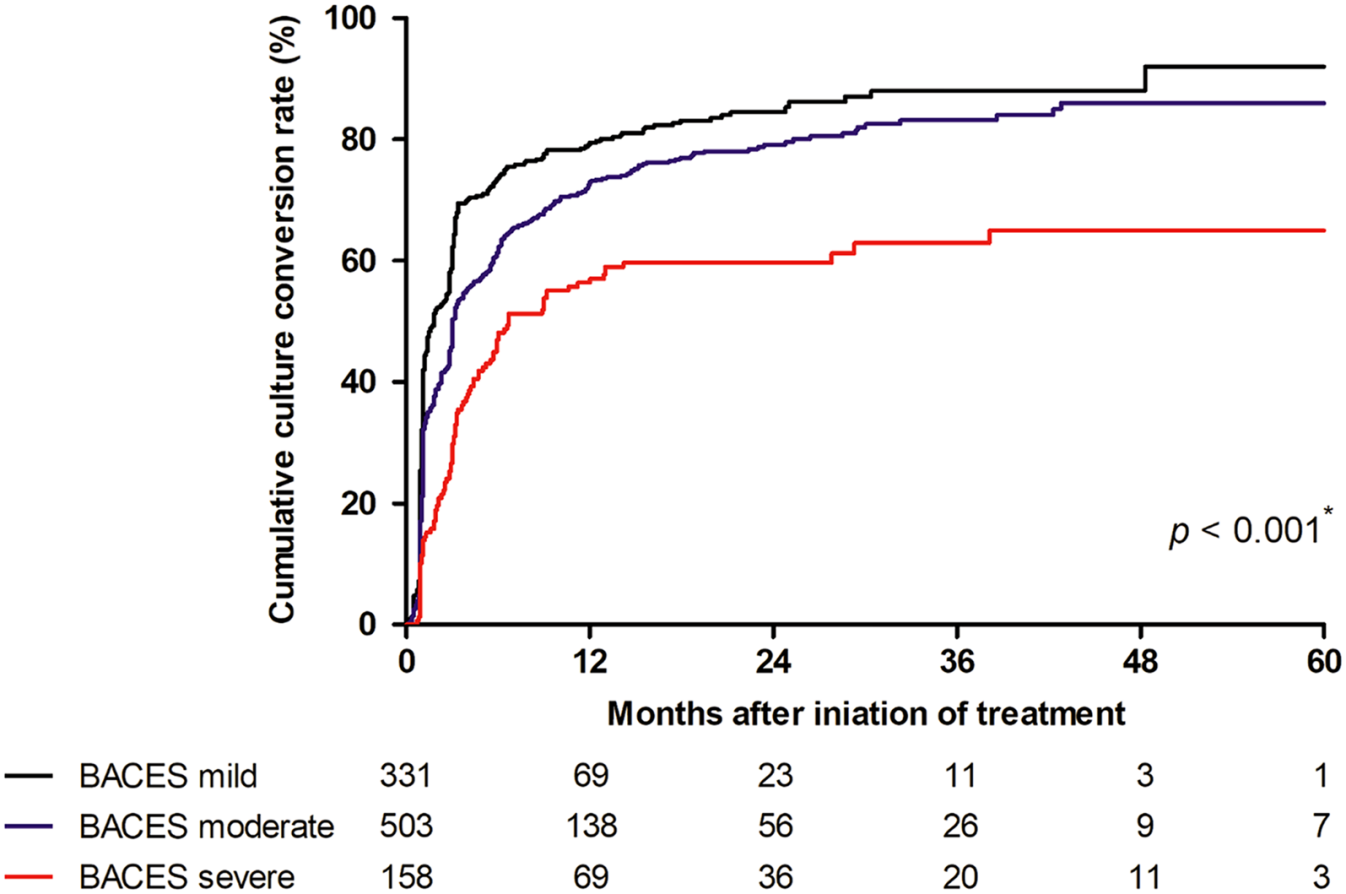
Cumulative culture conversion rate according to BACES severity group.

Notably, the culture conversion rates significantly decreased with an increase in BACES severity (*p* < 0.001). While 85% (282/331) of the mild group and 80% (403/503) of the moderate group achieved culture conversion, only 61% (97/158) of the severe group had culture conversion at the end of treatment. The difference in culture conversion rate was statistically significant between severe and mild/or moderate group (but not between mild and moderate group). The log-rank test indicated that the differences in the cumulative culture conversion among the BACES severity groups were significant (Fig. 2). In addition, the median time to culture conversion was significantly longer in the severe group, which had a median of 3.1 months (IQR 1.5–5.9), compared to the mild or moderate group.

### Treatment outcomes according to treatment modalities in the BACES mild group

**Figure 3:**
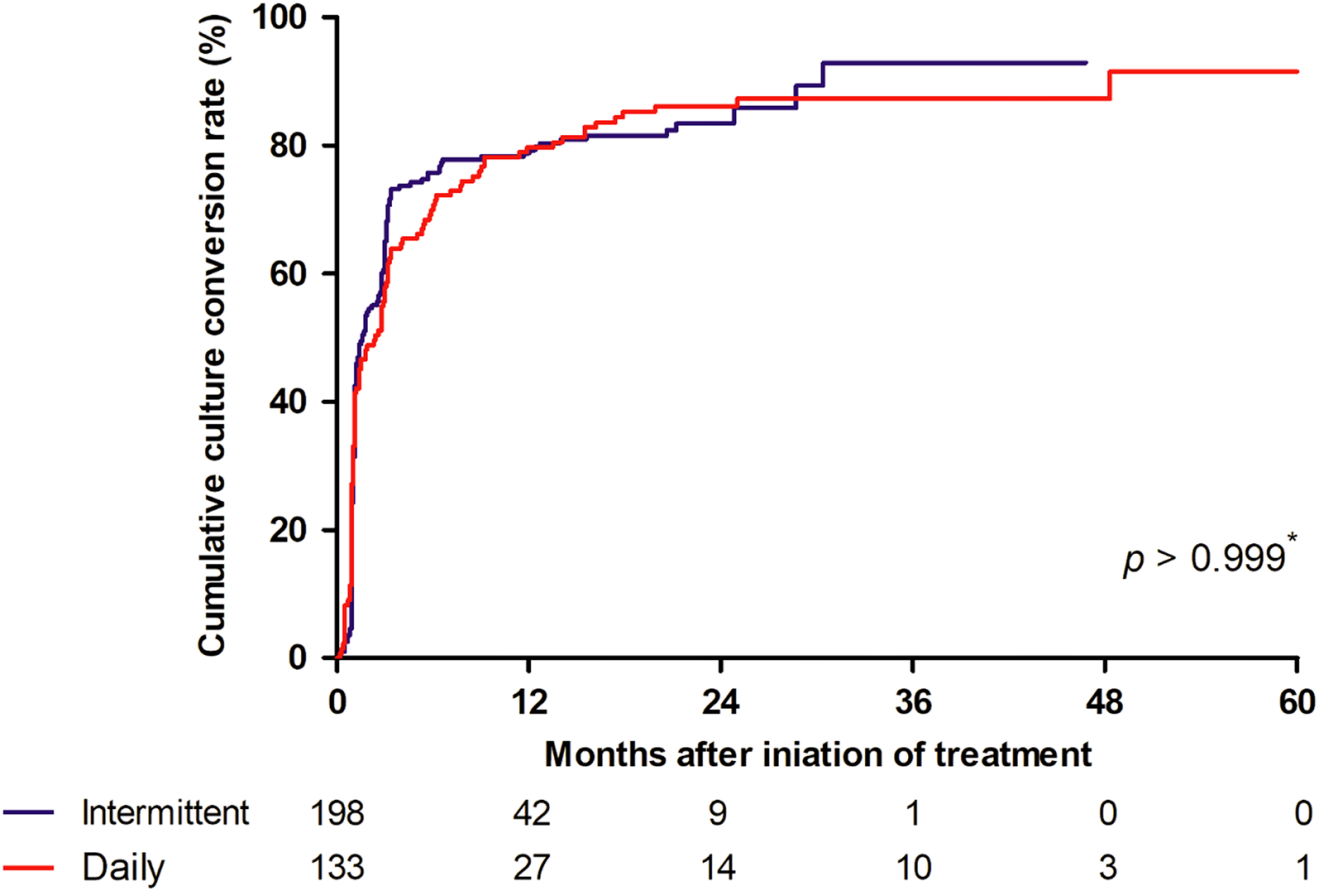
Cumulative culture conversion rate according to treatment modalities in the BACES mild group (n = 331).

Regarding treatment modalities, 60% (198/331) of the BACES mild group received intermittent treatment, instead of daily treatment (Supplementary Table 1). In the BACES mild group, 84% (166/198) of the intermittently and 87% (116/133) of the daily-treated patients had culture conversion, with no statistically significant difference (*p* = 0.396). The cumulative culture conversion rate according to the treatment modalities in the mild group also did not differ (Fig. 3). Culture conversion rates according to BACES parameters and treatment modalities in the BACES mild group did not significantly differ (Table 3).

**Table 3:** Culture conversion rate according to BACES parameters and treatment modalities in the BACES mild group.

	Total	Culture conversion, yes	Culture conversion, no	*p*-value
Intermittent treatment (n = 198)	n = 198	n = 166	n = 32	
BMI < 18.5 kg/m^2^	11/198 (6)	6/166 (4)	5/32 (16)	0.018
Age ≥ 65 years	13/198 (7)	12/166 (7)	1/32 (3)	0.697
Cavity	0/198 (0)	0/166 (0)	0/32 (0)	–
Elevated ESR*	88/198 (44)	75/166 (45)	13/32 (41)	0.635
ESR, mm/h	19 (10–33)	19 (11–32)	17 (8–35)	0.531
Sex, male	21/198 (11)	17/166 (10)	4/32 (13)	0.754
Daily treatment (n = 133)	n = 133	n = 116	n = 17	
BMI < 18.5 kg/m^2^	2/133 (2)	2/116 (2)	0/17 (0)	> 0.999
Age ≥ 65 years	4/133 (3)	3/116 (3)	1/17 (6)	0.425
Cavity	30/133 (23)	24/116 (21)	6/17 (35)	0.214
Elevated ESR*	47/133 (35)	42/116 (36)	5/17 (29)	0.787
ESR, mm/h	17 (12–27)	18 (12–28)	15 (9–24)	0.205
Sex, male	9/133 (7)	9/116 (8)	0/17 (0)	0.603

Data are presented as n (%).

*BACES* BMI, age, cavity, ESR, and sex; BMI, body mass index, *ESR* erythrocyte sedimentation rate.

*Elevated ESR: > 15 mm/h in men and > 20 mm/h in women.

### Effect of treatment modalities on culture conversion in the BACES mild group

We evaluated factors associated with culture conversion in the BACES mild group, including treatment modalities as a variable (Table 4). In our institution, intermittent therapy was performed for non-cavity NB MAC-PD patients from January 2011 onwards. However, since January 2011, daily therapy was still initiated for patients in the BACES mild group when certain criteria were met, as detailed in Supplementary Fig. 1. Of the 66 patients who treated daily therapy since January 2011, 27 patients had cavitation, 29 had radiological deterioration in CT, 8 had hemoptysis, and 2 had positive AFB smears. In univariable analysis, intermittent treatment was not associated with culture conversion (unadjusted hazard ratio 0.97; confidence interval 0.76–1.23; *p* = 0.788), and nor were comorbidity, etiology, use of aminoglycoside or clofazimine, or positive sputum AFB smear. After multivariable adjustment, intermittent antibiotic therapy did not negatively impact culture conversion (adjusted hazard ratio 1.08; confidence interval 0.83–1.41; *p* = 0.578).

**Table 4:** Factors associated with culture conversion in the BACES mild group (n = 331).

Variables	Culture conversion (n = 282)	Univariate		Multivariable	
		Unadjusted HR (95% CI)	*p*-value	Adjusted HR (95% CI)	*p*-value
**Comorbidity**					
Previous pulmonary tuberculosis	82 (29)	0.90 (0.69–1.16)	0.405	0.95 (0.72–1.25)	0.711
Obstructive pulmonary disease	14 (5)	1.41 (0.82–2.42)	0.210	1.50 (0.86–2.61)	0.156
Lung cancer	4 (1)	1.58 (0.59–4.24)	0.366	1.58 (0.58–4.32)	0.376
Diabetes mellitus	13 (5)	1.18 (0.68–2.06)	0.560	0.96 (0.53–1.76)	0.898
Chronic heart disease	10 (4)	1.56 (0.83–2.93)	0.172	1.58 (0.80–3.12)	0.184
**Etiology**					
*Mycobacterium avium*	173 (61)	Reference		Reference	
*Mycobacterium intracellulare*	109 (39)	0.99 (0.78–1.25)	0.911	0.95 (0.75–1.22)	0.696
Use of aminoglycoside injection*	25 (9)	0.79 (0.52–1.20)	0.270	0.84 (0.53–1.32)	0.440
Use of clofazimine	4 (1)	0.36 (0.13–0.97)	0.044	0.41 (0.15–1.12)	0.081
**Treatment modalities**					
Daily treatment	116 (41)	Reference		Reference	
Intermittent treatment	166 (59)	0.97 (0.76–1.23)	0.788	1.08 (0.83–1.41)	0.578
Positive sputum AFB smear	81 (29)	0.84 (0.65–1.08)	0.172	0.89 (0.68–1.17)	0.369

Data are presented as n (%) or median (interquartile range).

*BACES* body mass index, age, cavity, erythrocyte sedimentation rate, and sex, *HR* hazard ratio, *CI* confidence interval, *AFB* acid-fast bacilli.

*Patients who received aminoglycoside injection for > 3 months.

## Discussion

In the present study, we showed that a lower BACES severity in MAC-PD patients is associated with a higher culture conversion rate and shorter latency to conversion after treatment. In addition, intermittent antibiotic treatment did not affect the culture conversion rate in the mild disease group. Our findings suggest that daily and intermittent antibiotic therapy may have equivalent efficacy in mildly affected MAC-PD patients and that an intermittent treatment strategy may be useful to reduce pill burden in mild MAC-PD disease. Thus, our data imply that treatment response can be predicted based on disease severity and that therapeutic strategies can also be individualized according to the severity of MAC-PD.

That is, in BACES mild group, intermittent therapy can have the similar treatment response to daily treatment, and prolonged treatment can be beneficial for some patients who did not response to 12 months of treatment.

Notably, treatment outcomes, defined as negative sputum culture conversion rate, differed significantly among the MAC-PD patient groups stratified by BACES severity. Originally, the BACES severity scoring system was based on 5 year survival rates. Notably, based on our current findings, microbiological response can also be predicted using the BACES score. Several clinical or microbiological characteristics were previously identified as factors associated with culture conversion or treatment success^12,17–21^. In particular, previous studies found that a poor microbiological response was associated with fibrocavitary (FC) disease, positive AFB smear, and previously treated NTM^12,18,19^. However, there were some differences in the factors in each study, and no study showed analysis that could be applicable to real-world practice by composing individual factors comprehensively. An important cause of this limitation is the lack of indicators to assess the severity of MAC-PD. The heterogeneity in outcomes reported in previous meta-analysis is also presumed to be due to these above mentioned limitations^7,8,22^. In these contexts, BACES or other severity scoring systems may help in predicting treatment outcomes or strategies for MAC-PD patients.

Another important finding of our study was that intermittent antibiotic treatment did not have a negative effect on culture conversion at the end of treatment in the mild BACES MAC-PD group. These findings may enable the more specific selection of target populations that would benefit from intermittent therapy with low pill burdens, instead of daily treatment. For treating MAC-PD, previous cohort studies found that intermittent therapy was effective for treating MAC-PD patients with the noncavitary NB form of disease^12,14,23,24^. However, in real-world practice, there are some cases in which distinguishing between “advanced” and mild disease can be difficult. Some patients have severe bronchiectasis or destruction of the lung parenchyma without cavities, whereas others have only small cavitary nodules with mild bronchiectais. Notably, in our study, most (91%) of the mild BACES group did not have a cavity. Thus, our data implies that mild MAC-PD can be categorized by integrating several indicators such as BMI, age, and ESR, in addition to cavities. However, in the present study, no information was available regarding treatment outcome in the mild BACES and intermittent treatment group with cavitary lesions, so further studies regarding this issue are needed.

Interestingly, in the severe group, the culture conversion rate did not significantly increase even when the treatment period was extended to more than 12 months, whereas in the mild group, some additional patients (14/63, 22%) achieved culture conversion after 12 months of treatment. These findings suggest that, in patients with severe MAC-PD, when the treatment response is poor, such as persistent culture positivity, worsening of systemic symptoms, exacerbation of the chest CT lesion, and persistently high bacterial burden, it may be necessary to change the antibiotic regimen at an early stage or other interventions may be required. Conversely, in the mild group, some MAC-PD patients who did not achieve culture conversion even after 1 year of treatment could potentially achieve culture conversion if treatment were extended. These data may suggest that the treatment of refractory disease or the timing of regimen change should be determined differently depending on the severity of MAC-PD. In addition, in our study, the time from treatment to culture conversion was shorter among patients with mild disease than among those with either moderate or severe disease, suggesting that eventually the overall treatment period may be shortened in mild cases of MAC-PD. However, no studies have yet adjusted the duration of treatment for MAC-PD according to disease severity. Nevertheless, it will be important to determine whether some patients might benefit from a shorter treatment period without negatively affecting prognosis.

There are some limitations to our study. First, because it was conducted at a single referral centre, our data may not be generalizable to other geographic areas. Second, this study may have included unexpected biases due to the exclusion of patients in whom the treatment method was changed or who were treated for less than 12 months, according to the patient’s condition. Third, the BACES scoring system has not been validated in many cohorts of patients. Fourth, in the moderate and severe groups categorized by BACES, the treatment outcomes of intermittent *versus* daily treatment could not be properly compared. As shown in Supplementary Table 1, 28% (141/503) of the moderate group and 6% (10/158) of the severe group received intermittent treatment, instead of daily treatment; however, although their culture conversion rate did not differ significantly according to treatment modalities (*p* = 0.451 for the moderate group and *p* = 0.090 for the severe group), this result may not be valid due to the large differences in the ratios between intermittent *versus* daily treatment among the two groups. Fifth, in our study, treatment outcome was defined as negative conversion, not microbiological cure, because there is a possibility that the microbiological cure may be affected by the differences in the duration of antibiotic maintenance between the comparison groups. Lastly, BACES severity was determined at the time of diagnosis rather than at the start of the antibiotics treatment. However, the median time interval between the diagnosis and the start of treatment was 55 days. Thus, the potential effect of the time interval is not expected to be significant.

In conclusion, we showed that a lower BACES severity score in MAC-PD patients is associated with a higher culture conversion rate and shorter latency to conversion after treatment. Also, intermittent antibiotic treatment in the mild disease group did not affect the culture conversion rate. These data suggest that treatment modalities with a lower antibiotic burden are worth considering, especially in mild cases of MAC-PD.

## Methods

### Study population

A total of 1059 consecutive patients diagnosed with MAC-PD between January 2002 and December 2020 were identified from the NTM Registry of Samsung Medical Center, a referral hospital in Seoul, South Korea. From January 2002 to December 2007, data were obtained from a retrospective cohort, and beginning in January 2008, data were obtained from an ongoing Institutional Review Board-approved prospective observational cohort (ClinicalTrials.gov Identifier: NCT00970801)^15^. Informed consent was obtained from all individual participants, and this study was approved by an Institutional Review Board at Samsung Medical Center. All methods were performed in accordance with the relevant guidelines and regulations. For analysis, we included only patients with NB or FC form after excluding patients with unclassified form. Patients were excluded from the study if they changed treatment modalities from daily to intermittent (n = 26) or intermittent to daily (n = 41). All patients met the diagnostic criteria for MAC-PD.

### BACES severity

The severity of MAC-PD was calculated using the BACES score (BMI < 18.5 kg/m^2^, age ≥ 65 years, presence of cavity, elevated ESR, and male sex, each one point)^16^. All patients were classified into three groups on the basis of the scored severity at the time of diagnosis: mild (0–1 point), moderate (2–3 points), and severe (4–5 points) (Fig. 1).

### Antibiotic regimen

The antibiotic regimens were based on American Thoracic Society guidelines^3,25^. All MAC-PD patients who started therapy received combination antibiotic therapy consisting of an oral macrolide (clarithromycin or azithromycin), ethambutol, and rifamycin (rifampicin or rifabutin)^18^. Regarding treatment modalities, all patients were treated with standard daily therapy before January 2011. After January 2011, most MAC-PD patients with non-cavitary NB MAC-PD were treated with intermittent therapy, three-times-weekly^14^. In some cases, the treatment modalities were changed at the discretion of the attending physician with consideration of the antibiotic toxicity and benefits, but patients who changed antibiotic administration method within 12 months after starting therapy were excluded from this analysis (Fig. 1). Aminoglycosides were additionally administered in patients with severe disease for the first several months. Oral clofazimine (100 mg once daily) was also added in refractory MAC-PD patients at the discretion of the attending physician^26^.

### Microbiological and radiological examinations

Sputum AFB smears and cultures were performed using standard methods at 1–3 month intervals. Specimens were cultured both on 3% Ogawa solid medium (Shinyang, Seoul, Korea) and in liquid broth medium in mycobacterial growth indicator tubes (MGIT; Becton, Dickinson and Co., Sparks, MD, USA). NTM species were identified using polymerase chain reaction-restriction fragment length polymorphism analysis or reverse-blot hybridization of the *rpoB* gene. Beginning in June 2014, species identification was conducted via nested multiplex polymerase chain reaction and a reverse-hybridization assay of the internal transcribed spacer region (AdvanSure Mycobacteria GenoBlot Assay; LG Life Sciences, Seoul, South Korea)^27,28^. The radiological form of MAC-PD was categorized as FC, cavitary NB, or non-cavitary NB form^18^.

### Treatment outcome

Treatment outcome, defined as sputum negative culture conversion, was assessed at 12 months after starting treatment and at the end of treatment based on the NTM-NET consensus statement^29^. Sputum negative culture conversion was defined as at least three consecutive negative sputum cultures after treatment, collected at least four weeks apart. The time of conversion was defined as the date of the first negative culture.

### Statistical analysis

Data are presented as numbers (percentages) for categorical variables and medians (IQR) for continuous variables. Continuous data were compared by the Mann–Whitney test or Kruskal–Wallis test, and categorical data were compared by the chi-squared test or Fisher’s exact test. Bonferroni's method was used for post hoc analysis. The Kaplan–Meier method was used to estimate the cumulative culture conversion rates of MAC-PD patients, and the log-rank test was used to compare the curves. A Cox proportional hazard regression analysis was used to identify factors associated with a culture conversion. All tests were two-sided, and a *p*-value < 0.05 was considered significant. All statistical analyses were performed using SPSS (IBM SPSS Statistics ver. 27, Chicago, IL, USA).

## Supplementary Information

Supplementary Information.

## Abbreviations

NTM-PD: Nontuberculous mycobacterial pulmonary disease
MAC: *Mycobacterium avium* complex
BMI: Body mass index
AFB: Acid-fast bacilli
ESR: Erythrocyte sedimentation rate
CT: Computed tomography
FC: Fibrocavitary
NB: Nodular bronchiectatic
CFZ: Clofazimine
IQR: Interquartile range
