# Supplementary material for: Treatment outcomes of Mycobacterium avium complex pulmonary disease according to disease severity
Source: Sci Rep. 2022 Feb 4;12:1970. doi: 10.1038/s41598-022-06022-z (PMC8816953; doi:10.1038/s41598-022-06022-z)

**[Supplementary data]**

**Treatment outcomes of Mycobacterium avium complex pulmonary disease according to disease severity**

Bo-Guen Kim^1^, Byung Woo Jhun^1^, Hojoong Kim^1^, O Jung Kwon^1^

^1^Division of Pulmonary and Critical Care Medicine, Department of Medicine, Samsung Medical Center, Sungkyunkwan University School of Medicine, Seoul, South Korea.

**Supplementary Table 1.** Culture conversion rate at the end of treatment according to treatment modalities in each BACES severity group

| BACES severity | Culture conversion | | | *p*-value |
| --- | --- | --- | --- | --- |
|  | Total | Intermittent treatment | Daily treatment |  |
| Mild | 282/331 (85) | 166/198 (84) | 116/133 (87) | 0.396 |
| Moderate | 403/503 (80) | 116/141 (82) | 287/362 (79) | 0.451 |
| Severe | 97/158 (61) | 9/10 (90) | 88/148 (59) | 0.090 |

Data are presented as n (%). Abbreviations: BACES, body mass index, age, cavity, erythrocyte sedimentation rate, and sex.

**Supplementary Figure 1.** Characteristics of study patients in the BACES mild group. Abbreviations: AFB, acid-fast bacilli; CT, computed tomography.


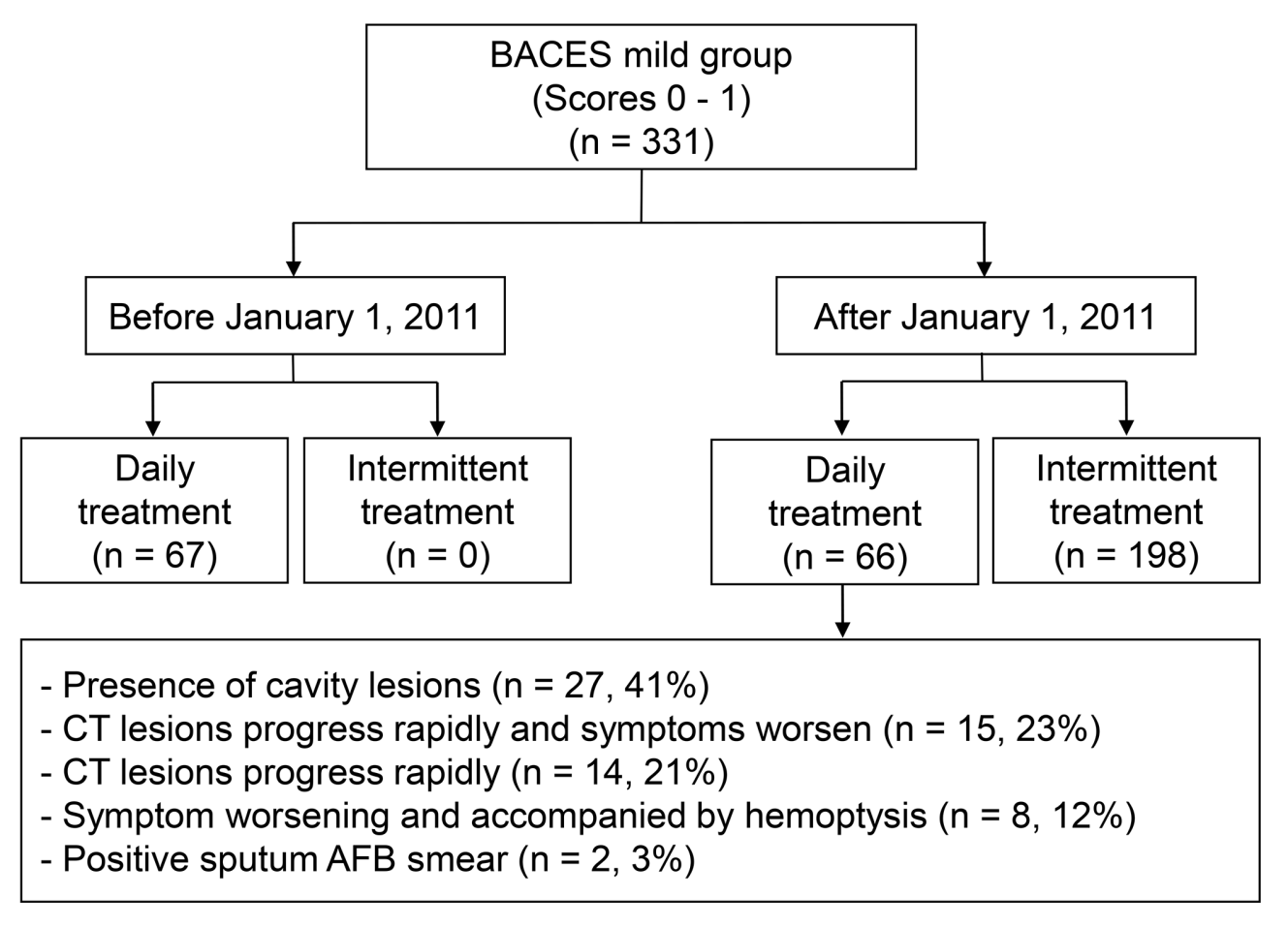

Supplement: Supplementary file 1 — Supplementary Information. [file 41598_2022_6022_MOESM1_ESM.docx]
